# Case report: Anti-ARHGAP26 autoantibodies in atypical dementia with Lewy bodies

**DOI:** 10.3389/frdem.2023.1227823

**Published:** 2023-08-03

**Authors:** Niels Hansen, Kristin Rentzsch, Sina Hirschel, Jens Wiltfang, Björn Hendrik Schott, Claudia Bartels, Claudia Lange, Caroline Bouter

**Affiliations:** ^1^Department of Psychiatry and Psychotherapy, University Medical Center Göttingen, Göttingen, Germany; ^2^Clinical Immunological Laboratory Prof. Stöcker, Groß Grönau, Germany; ^3^German Center for Neurodegenerative Diseases (DZNE), Göttingen, Germany; ^4^Neurosciences and Signaling Group, Department of Medical Sciences, Institute of Biomedicine (iBiMED), University of Aveiro, Aveiro, Portugal; ^5^Leibniz Institute for Neurobiology, University of Magdeburg, Magdeburg, Germany; ^6^Department of Nuclear Medicine, University Medical Center Göttingen, Göttingen, Germany

**Keywords:** autoimmunity, cognition, ARHGAP26 autoantibodies, dementia with Lewy bodies, Alzheimer's dementia

## Abstract

**Background:**

Dementia with Lewy bodies (DLB) is the second most common type of neurodegenerative dementia. Here, we report a case of dementia associated with anti-Rho-GTPase-activating protein 26 (ARHGAP26) autoantibodies, which have never been previously linked to DLB.

**Methods:**

We describe the case of a 78-year-old man who underwent cerebrospinal fluid (CSF) analysis, magnetic resonance imaging (MRI), ^18^F-fluorodesoxyglucose positron emission tomography (FDG-PET), and a detailed neuropsychological evaluation.

**Results:**

The patient presented with mild dementia syndrome associated with extrapyramidal symptoms. Neuropsychological testing revealed impaired cognitive flexibility, figural memory, and verbal memory. Fluctuating cognitive abilities with deficits in attention-executive dysfunction and visuoconstruction also developed over time. A brain MRI showed reduced biparietal and cerebellar brain volume with generalized accentuation of the outer CSF spaces. The patient's CSF revealed anti-ARHGAP26 autoantibodies, which were also detectable in serum. In the differential complementary imaging diagnosis at 2 years, an FDG-PET revealed decreased occupancy of the posterior cingulum and precuneus. Although the FDG-PET, MRI, and clinical findings were potentially consistent with Alzheimer's disease, negative amyloid biomarkers in the CSF made an AD diagnosis highly unlikely. Single photon emission computed tomography (SPECT) with [(123)I] N-omega-fluoropropyl-2beta-carbomethoxy-3beta-{4-iodophenyl}nortropane ([(123)I]FP-CIT) showed right-sided predominance, reduced dopamine transporter uptake in the putamen, consistent with a positive indicative biomarker finding typical of DLB. Considering the clinically probable DLB associated with the two core features of Parkinsonism and fluctuating cognition with deficits in attention, supported by an abundant tracer uptake in the right putamen and lower uptake in the left putamen on 123I-FP-CIT-SPECT as an indicative biomarker, we started an antidementia drug using a cholinesterase inhibitor.

**Conclusions:**

Our report shows that atypical DLB may be associated with anti-ARHGAP26 autoantibodies, although their role and significance in the pathogenesis of DLB are unknown. However, it has to be mentioned that it is also possible that antibody-specific synthesis of anti-ARHGAP26 autoantibodies is a hallmark of a rare autoimmune disease that may cause the clinical and laboratory features involving altered dopamine transporter uptake on 123I-FP-CIT-SPECT, dementia, and mild Parkinson's symptoms rather than idiopathic DLB with only two core DLB features and inconsistent cognitive and imaging findings. Further research is needed to investigate the role of these autoantibodies in different dementias, particularly in DLB and mixed DLB-AD types.

## 1. Introduction

Dementia with Lewy bodies (DLB) is the second most common form of dementia, and its pathophysiology is associated with abnormal aggregation of alpha-synuclein. DLB is usually idiopathic, although genetic factors such as apolipoprotein ε4 and the glucocerebrosidase gene may contribute to Lewy body pathology (Kaivola et al., 2022). Increased levels of autoantibodies against glial antigens have recently been detected in DLB compared with other forms of dementia that are partially attributable to tauopathies, such as Alzheimer's disease and frontotemporal dementia (Maetzler et al., 2011). However, this interesting pathway for a potential autoantibody biomarker in early DLB has not been further explored. Other autoantibodies identified in patients with DLB have been reported, such as anti-NH2-terminal α-enolase (anti-NAE) along with elevated levels of anti-glutamate receptor ε2 subunit (GluRε2) antibodies in a patient with DLB (Ikura et al., 2015). However, the role of autoantibodies in DLB remains unclear. Here, we describe a DLB patient in whom we detected anti-Rho-GTPase-activating protein 26 (anti-ARHGAP26) autoantibodies. Anti-ARHGAP26 is a GTPase-activating protein (Hildebrand et al., 1996). It inhibits the activity of Rho GTPases by enhancing their hydrolytic ability (Zhang et al., 2021). By altering the activity of Rho-GTPases, anti-ARHGAP26 autoantibodies could exert effects on the intracellular signaling cascades that influence signal transduction in various cells, i.e., cell migration and cell growth (Chen et al., 2019). In addition, the blockade of anti-ARHGAP26 autoantibodies impairs Rho GTPases, which are important signaling proteins critical for synaptic connectivity (Ramakers, 2002) and plasticity (Zhang et al., 2021). Altered Rho signaling by anti-ARHGAP26 autoantibodies can trigger cognitive dysfunction in humans (Ramakers, 2002). Cognitive impairment has been associated with such anti-ARHGAP26 autoantibodies and is mainly detected in diseases entailing cerebellar ataxia associated with immunopathology in cerebellar structures (Doss et al., 2014; Jarius and Wildemann, 2015; Bartels et al., 2018). Other clinical features associated with anti-ARHGAP26 autoantibodies include vertigo, cerebellar atrophy, depression, and dysarthria (Doss et al., 2014; Jarius and Wildemann, 2015; Wallwitz et al., 2017). In addition, a patient has recently been described with motor symptoms such as apraxia, rigor, tremor, dysarthria, stimulus-sensitive myoclonic twitching of all limbs, mild cognitive impairment, and a verbal learning disability associated with anti-ARHGAP26 autoantibodies (Schegk et al., 2023). Anti-ARHGAP26 autoantibodies have been shown to be similarly prevalent in neuropsychiatric disorders as in healthy controls (Dahm et al., 2014), suggesting that more evidence is needed to support the potential pathogenicity of these autoantibodies. Clinically apparent cognitive dysfunction associated with anti-ARHGAP26 autoantibodies is not new, but to our knowledge, this is the first report documenting anti-ARHGAP26 autoantibodies in dementia disorders such as DLB.

## 2. Case report

**Figure 1:**
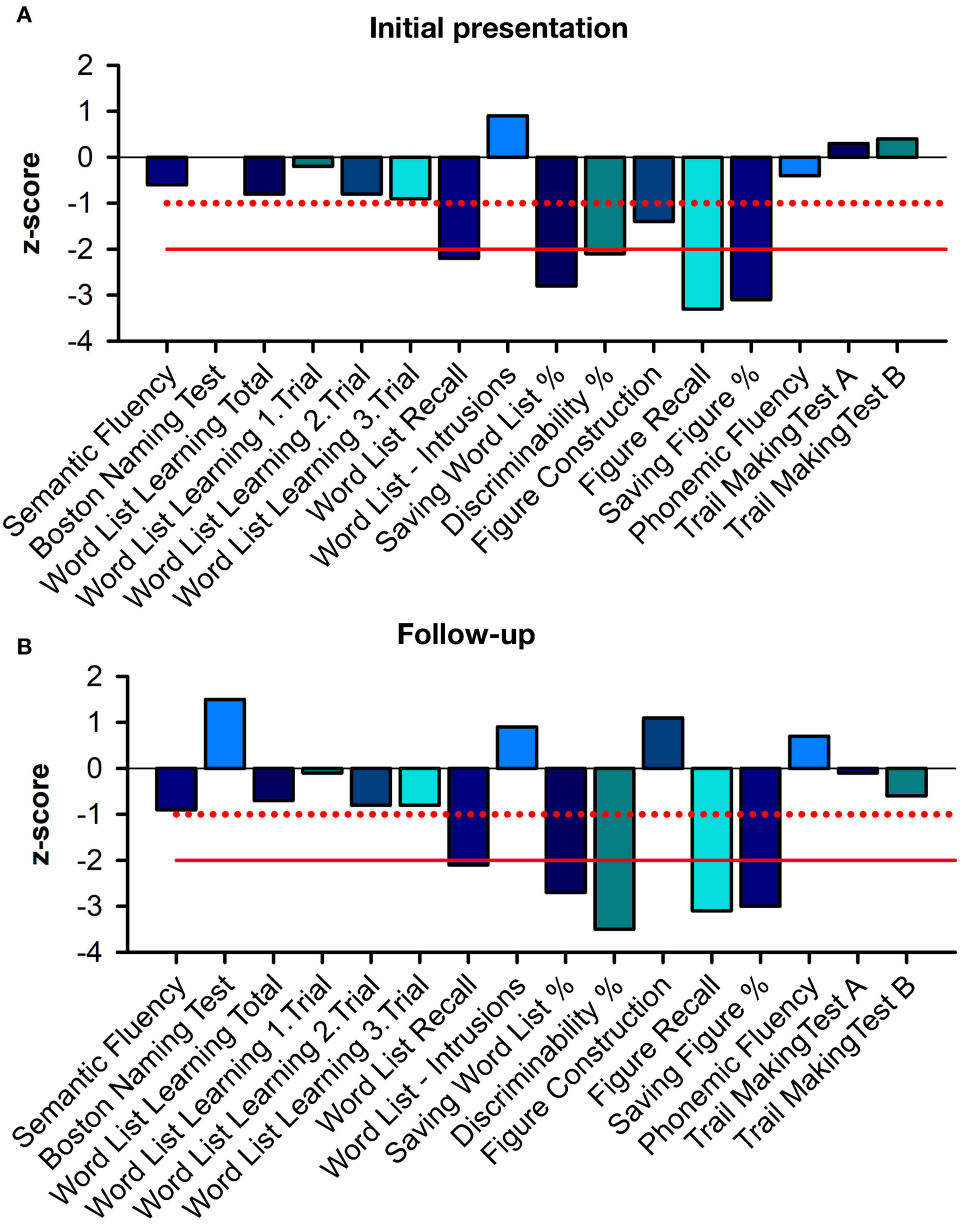
Neuropsychological profile at initial presentation and follow-up. **(A)** shows the neuropsychological profile at the initial presentation, and **(B)** at the follow-up. A slight improvement in figure construction and the Boston naming test at follow-up can be seen. The red solid line indicates the twofold standard deviation, while the dashed red line means the single standard deviation.

**Figure 2:**
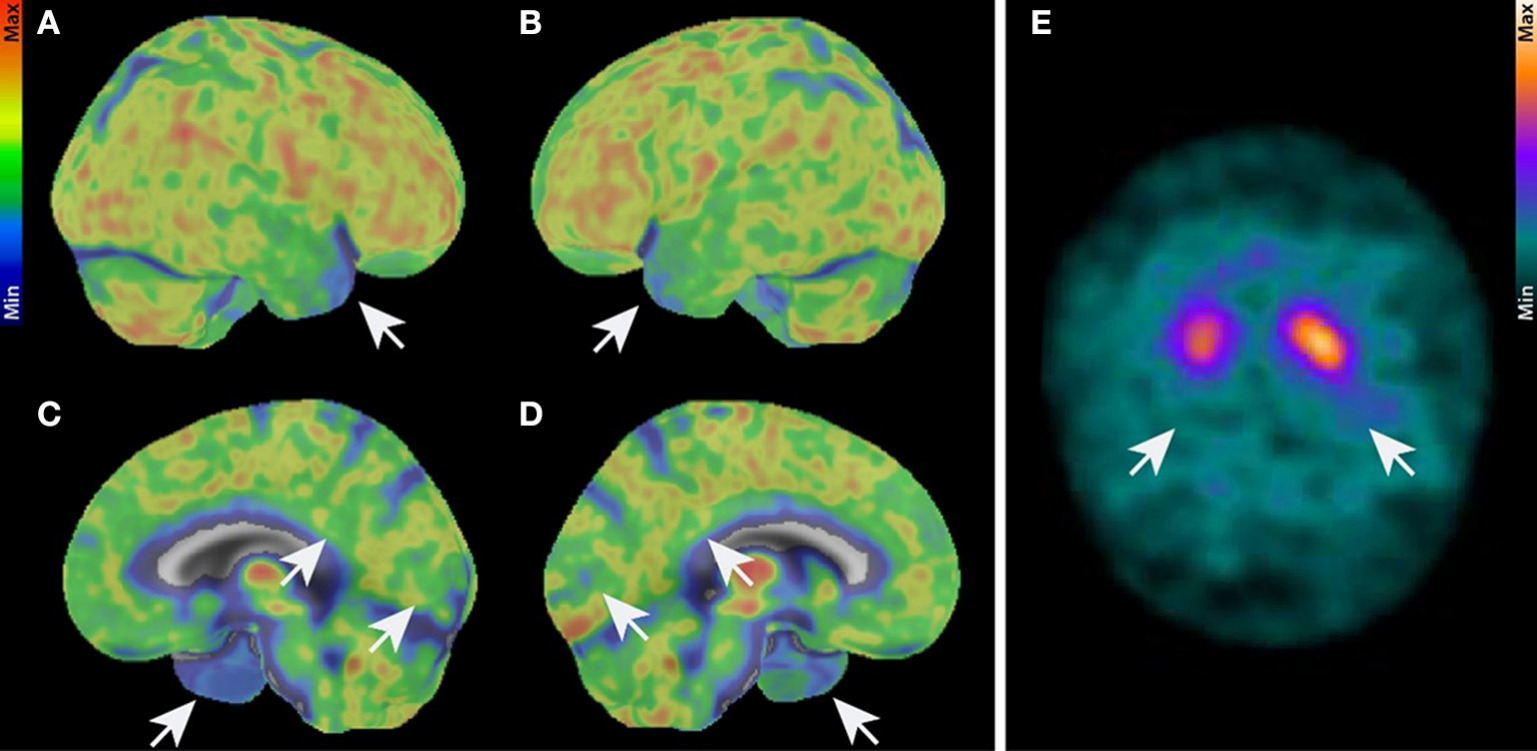
Imaging. **(A–D)** 18F-FDG-PET. Brain hemisphere surface projections of ^18^F-FDG- uptake in right lateral **(A)**, left lateral **(B)**, right medial **(C)**, and left medial **(B)** views show reduced tracer uptake in the temporal cortex, the posterior cingulate, precuneus, and slightly reduced uptake in the visual cortex (white arrows). **(E)** ^123^I-FP-CIT-SPECT in axial view showing abundant tracer uptake in the right putamen and lower uptake in the left putamen (white arrows). 123I-FP-CIT-SPECT, Single photon emission computed tomography with [(123)I]; N-omega-fluoropropyl-2betacarbomethoxy-3beta-{4-iodophenyl}nortropane; 18F-FDG-PET, 18F fluorodesoxyglucose positron emission tomography.

A 78-year-old male patient presented with fluctuating cognitive dysfunction. He reported a slowly insidious memory impairment that had been present for 5 years. He also suffered from temporal and spatial orientation problems and stated that he found it difficult to retain information from conversations. His medical history indicated arterial hypertension. There was no evidence of a manic or depressive episode in this patient's history. He lived with his wife, had completed primary school in 8 years, and then attended commercial school for 3 more years. He was first a professional soldier, then worked at a company in Göttingen, and was now retired. He had one sister and no children; his mother died of Parkinson's disease at the age of 79. His psychiatric examination revealed major memory and retention deficits, in addition to poor spatial and temporal orientation. A neurological examination revealed Parkinsonian symptoms, and temporal-spatial impairment, and memory deficits, manifested by left limb tremors and an inexhaustible glabellar reflex. There was no evidence of cerebellar ataxia. His Mini-Mental Status Examination (MMSE) was 25/30 in 2020, and he scored 1 out of 30 on the Geriatric Depression Scale. A detailed neuropsychological examination revealed impaired cognitive flexibility, impaired figural memory, verbal memory impairment in encoding, delayed recall of complex verbal content, and consolidation of verbal non-associated information (Figure 1A). Other parameters such as semantic word fluency, phonemic word fluency, confrontation naming, visuomotor coordination, cognitive processing speed, switching ability, action planning, working memory, encoding of verbal non-associated information, visuoconstructive skills, and visual-spatial perception were inconspicuous considering his education. In this 2022 examination (Figure 1B), we noted a worse mental performance in cognitive fluency, switching ability, and immediate memory span compared to the 2020 examination. Furthermore, attention and executive functions were worse in 2022 than in 2020. In particular, the formal lexical category difference and a change in the semantic category on the “Regensburger Wortflüssigkeits Test” were pathological. We also found deficits in visual-constructive skills that were not present in 2020. The Shulman clock-drawing test in 2020 showed no impairment in visual-constructive skills with a score of 1, but was impaired 2 years later with a score of 3, indicating deficient visual-spatial skills. In addition, the patient's constructive abilities on a CERAD drawing were pathological, so overall there was little improvement but deterioration in various cognitive domains, findings consistent with fluctuations in cognitive performance in DLB patients and in those with neurodegenerative dementia. Daily living limitations were less pronounced, according to his wife, with a Bayer Activities of Daily Living scale score of 4.8. Overall, this corresponds to mild dementia syndrome, according to clinical scoring. The patient's initial brain MRI showed reduced biparietal and cerebellar brain volume with generalized enhancement of the outer CSF spaces. Parietal brain atrophy on an MRI could be consistent with degenerative dementia. His neurologic examination revealed no cerebellar syndrome, although cerebellar atrophy was present in the MRI. His CSF and serum revealed anti-ARHGAP26 autoantibodies. We determined the antibody-specific index (ASI) of ARHGAP26 autoantibodies, which was 16.32, indicating a synthesis of ARHGAP26-specific autoantibodies. Neither IgG synthesis nor pleocytosis were detected in the CSF (Supplementary Table 1). Ptau181 was slightly elevated in his first CSF examination (Supplementary Table 1) but not in the second one. Total tau protein was pathologically elevated only in his second CSF examination (Supplementary Table 1). The ratio of amyloid β42 to amyloid β40 (Aβ 42/40) and Aβ 42 in CSF was not pathologically low (Supplementary Table 1). We searched for various neuronal autoantibodies in the clinical immunology laboratory of Prof. Stöcker. Autoantibody detection methods included BIOCHIP mosaics with brain tissue and cell-based assays incorporating autoantibodies against membrane and intracellular antigens. The spectrum of neuronal autoantibodies we studied was as follows: anti-amphiphysin, -glutamic acid decarboxylase 65 (GAD65), -contactin-associated protein-2 (CASPR2), -CV2, -dipeptidyl peptidase-like protein 6 (DPPX), -gamma-aminobutyric acid 1/2 (GABA1/2), -HuD, leucine-rich inactivated glioma protein I1 (LGI1), -Ma2, -N-methyl-D-aspartate receptor (NMDAR), -Ri, -SOX1,-Tr, -Yo, -Zic4, and -α-amino-3-hydroxy-5-methyl-4-isoxazolepropionic acid receptor 1/2 (AMPAR1/2) autoantibodies. Cell destruction markers were slightly abnormal according to the analysis of the Laboratory of Clinical Neurochemistry and Dementia Diagnostics of the Department of Psychiatry and Psychotherapy, University Hospital Erlangen, which revealed transient but elevated phosphorylated tau protein 181 (ptau181) and elevated total tau protein (t-tau) (Supplementary Table 1). In the differential diagnostic imaging performed at 2 years, an ^18^F-FDG-PET showed reduced tracer uptake in the temporal cortex, posterior cingulate, and precuneus, consistent with AD pathology (Figures 2A–D). Mild hypometabolism was also detected in the visual cortex, indicating a possible diagnosis of DLB. A ^123^I-FP-CIT-SPECT showed abundant tracer uptake in the right putamen and lower uptake in the left putamen (Figure 2E). Considering the clinical likelihood of DLB with core Parkinsonian symptoms and cognitive fluctuations supported by the nigrostriatal deficit in ^123^I-FP-CIT-SPECT as a validated indicative biomarker, we started the patient on the antidementia drug rivastigmine. Other core clinical features of DLB, such as REM sleep behavior disorder and visual hallucinations, were not present. However, it is also conceivable that he had a mixed pathology between AD and DLB based on the FDG-PET evidence. The ^18^F-FDG-PET showed a pattern that is more likely to be found in AD patients, as a recent publication reported that patients with cognitive impairment and AD pathology are more likely to reveal impaired medial temporal and cingulate metabolism, whereas these structures are unaffected in DLB patients (Kantarci et al., 2021). However, FDG-PET currently serves only as a supportive biomarker in AD, indicating neuronal injury. An FDG-PET is also only a supportive biomarker in DLB (McKeith et al., 2017), in contrast to the indicative biomarker showing abundant tracer uptake in the right putamen and lower uptake in the left putamen on 123 CIT SPECT. As precuneus hypometabolism is also observed in some patients with DLB (Liu et al., 2017), DLB is also conceivable when precuneus hypometabolism is apparent. AD pathology is also diagnosed according to AT(N)(AT(N) = β-amyloid deposition, pathological tau, and neurodegeneration) classification of Jack et al. (2018), in which brain amyloid positivity must be verified by CSF or amyloid PET. It is therefore unlikely that our patient suffered from AD. His ptau181 was not consistently elevated over time. DLB pathology was more likely; therefore, AD pathology without proven brain amyloidosis was unlikely. Our patient's clinical presentation of cognitive deficits was not typical of DLB, but they may be consistent with cognitively fluctuating attention-executive functions, his defective visuoconstructive abilities, reduced cognitive flexibility, and impaired delayed recall (criteria: McKeith et al., 2017; for impaired recall in DLB, Tagawa et al., 2015), although his visuoconstructive functions were not impaired. Thus, because of the correlating anti-ARHGAP26 autoantibodies, we suggest a diagnosis of atypical DLB according to two fulfilled core features (fluctuating cognition, Parkinsonism) and one positive indicative biomarker, namely confirmed pathological I123 CIT-SPECT. Other neurodegenerative diseases or dementias are unlikely because none of their typical clinical features were present, thus excluding multisystem atrophy, frontotemporal lobar degeneration, and progressive supranuclear palsy. The patient's cognitive symptoms preceded his motor symptoms, thus making dementia due to Parkinson's disease unlikely. Rivastigmine as a patch was not tolerated, so we changed the patch and started him on donepezil 5 mg/d. As he has undergone no further follow-up, we have no further access to data on his neuropsychological profile or how he is tolerating the acetylcholine esterase inhibitor donepezil. During the disease course, he should undergo oncologic screening such as whole-body positron emission computed tomography because a paraneoplastic syndrome may precede a tumor by years, as in patients with Rho GTPase-activating protein 10 (Jarius et al., 2022). In addition, anti-ARHGAP26 autoantibodies associated with isolated cognitive impairment have also been reported to be associated with tumors (Bartels et al., 2018).

## 3. Discussion

Our report demonstrates anti-ARHGAP26 autoantibodies in a male patient with significant Lewy body pathology based on two core clinical features (fluctuating cognitive deficits with deficits in attention, and Parkinsonism) and an indicative biomarker (reduced dopamine transporter uptake) according to the McKeith criteria (McKeith et al., 2017). Mixed DLB-AD pathology is conceivable based on the patient's MRI, clinical, and PET findings, although this diagnosis is unlikely due to the absence of AD pathology (in particular, no positive proxy for brain amyloidosis) in the CSF according to the internationally accepted AT(N) diagnostic criteria (Jack et al., 2018) and the International Working Group criteria (Dubois et al., 2021) for AD. The frequent reports of cognitive impairment associated with anti-ARHGAP26 autoantibodies suggest that anti-ARHGAP26 autoantibodies may reflect additional neuroinflammation that is not part of DLB or is secondary to neurodegeneration. However, it is unclear whether a certain subset of DLB patients have an inflammatory phenotype involving autoantibodies such as anti-ARHGAP26 autoantibodies or autoantibodies to glial antigens, and whether there are other features that characterize such patients. The broad spectrum of anti-ARHGAP26 autoantibodies, ranging from cerebellar syndrome to cognitive dysfunction and psychosis, may indicate different regional brain involvement depending on which part of the brain the anti-ARHGAP26 autoantibodies were deposited. The patient's FDG-PET scan showed marked hypometabolism in the precuneus and posterior cingulate cortex, suggesting atypical features of DLB. Our detection of autoantibodies to ARHGAP26 in both his CSF and serum, together with the brain abnormalities on FDG-PET and MRI, suggested probable autoimmune-related cognitive impairment. However, he did not meet the Graus criteria for autoimmune encephalitis (Graus et al., 2016) because DLB is a concurrent cause of cognitive decline. A recent study found marked T cell recruitment without relevant microglial activation in 30 postmortem confirmed DLB cases compared to controls (Amin et al., 2020). According to another review by this group, alpha-synuclein triggers inflammation and underlies AD pathology as another feature of inflammation in DLB. Another study (Gate et al., 2021) delivered circumstantial evidence for autoimmunity associated with neurodegeneration in DLB patients, as CD4+ T cells were detected in postmortem brains near Lewy bodies. Since the AHRGAP26 protein is an intracellular target, this is not inconsistent with a T-cell-mediated pathophysiological mechanism, as intracellular autoantibodies are often found in association with a T cell mechanism in autoimmune encephalitis (Bien et al., 2012). Furthermore, it is tempting to speculate that anti-ARHGAP26 autoantibodies may interfere with the formation of figural memory in our case by blocking the ARHGAP26 target by autoantibodies, resulting in disrupted synaptic plasticity relevant for enabling figural memory. As this exact mechanism is unknown, it deserves further investigation in the future.

### 3.1. Limitations

While it is unclear whether anti-ARHGAP26 autoantibodies are merely an epiphenomenon, evidence from CSF and serum antibodies argues against this assumption. Anti-ARHGAP26 autoantibodies are detected at low frequencies in affective disorders (2.27%) and stroke (2.26%) compared with healthy controls (0.88%). However, no such autoantibodies have been so far observed in association with personality disorders, addictive disorders, developmental disorders, or neurodegenerative disorders (Daguano Gastaldi et al., 2022). It is therefore highly likely that anti-ARHGAP26 autoantibodies will eventually be found to correlate with various clinical conditions associated with affective disorders, cerebral ischemia, or cerebellar ataxia, but further investigation is needed to provide evidence of brain inflammation and neuronal damage, suggesting the clinical relevance of these autoantibodies. Because of the association between anti-ARHGAP26 autoantibodies and his dementia syndrome, it is quite conceivable that some of his cognitive symptoms are caused by anti-ARHGAP26 autoantibodies. However, it is also possible that this is an epiphenomenon associated with anti-ARHGAP26 autoantibodies and that the patient's cognitive impairment is merely coincident with anti-ARHGAP26 autoantibodies and not caused by them. This issue can only be conclusively clarified in larger cohort studies of patients presenting with cognitive impairment with or without DLB, and with or without anti-ARHGAP26 autoantibodies. Moreover, to better assess whether anti-ARHGAP26 autoantibodies do play a pathogenic role, it would have been helpful to determine whether activated CD4+ T cells were present in the CSF. CD4+ T cells can be elevated in autoimmune temporal lobe involvement (Hansen et al., 2022). Another limitation we should mention is our patient's memory impairment, as memory is usually preserved in DLB. It is also possible that anti-ARHGAP26 autoantibodies, as a rare autoimmune disease, may be the cause of the subject's clinical and laboratory features with altered dopamine transporter uptake, dementia, and mild Parkinsonian symptoms rather than idiopathic DLB (fulfilling only two core features and with inconsistent cognitive and imaging findings).

### 3.2. Conclusions

Our report potentially provides additional evidence for the possible autoimmune involvement of cerebrospinal ARHGAP26 antibodies in DLB. The merits of our case report lie in the novelty of the association between anti-ARHGAP26 autoantibodies and the presence of DLB. This evidence supports the increasingly observed phenomenon of neuronal autoantibodies in neurodegenerative diseases (Bastiaansen et al., 2023). However, no conclusions can be drawn currently regarding the frequency of detection of this autoantibody in DLB patients, as it may also be an incidental finding-a shortcoming of our report. Further studies will reveal whether anti-ARHGAP26 autoantibodies are present in DLB in individual patients and whether they may contribute relevantly to the pathophysiology of neurodegenerative dementia.

## Data availability statement

The raw data supporting the conclusions of this article will be made available by the corresponding author, without undue reservation.

## Ethics statement

The study involving human participants was reviewed and approved by Ethics Committee of the University Medical Center Göttingen. Written informed consent for participation was not required for this study in accordance with the national legislation and the institutional requirements. Written informed consent was obtained from the individual(s) for the publication of any potentially identifiable images or data included in this article.

## Author contributions

NH wrote the manuscript. All authors have read, edited, and agreed to the submitted version of the manuscript.
