# Supplementary material for: Case report: Anti-ARHGAP26 autoantibodies in atypical dementia with Lewy bodies
Source: Front Dement. 2023 Aug 3;2:1227823. doi: 10.3389/frdem.2023.1227823 (PMC11285637; doi:10.3389/frdem.2023.1227823)
Supplement: Supplementary file 1 [file Data_Sheet_1.pdf]

Table 1: Clinical and CSF data

PARAMETER	First presentation	20 month later	23 month later
**Demographic parameter**			
Age y	78	80	80
Age of onset y	73	73	73
**MMSE**	25/30	26/30	
**GDS**	01	01	
**B-ADL**	5.7	4.8	
**CSF parameter**			
Cell count (<5µl)	0	1	-
Lactate mmol/l	1.3	1.1	-
Albumin mg/L	169	151	-
Total protein mg/L	275	266	-
IgG mg/L	16.1	13.4	-
IgA mg/L	1.7	1.5	-
IgM mg/L	0.31	0.16	-
QAlbumin	3.9	3.9	-
QIgG	2	1.9	-
QIgA	0.76	0.84	-
QIgM	0.45	0.34	-
Measles AI	0.9	0.9	-
Rubella AI	0.8	0.8	-
VZV AI	0.9	1	-
Tau protein (<320pg/ml)	313	359	-
P Tau protein 181 (<50pg/ml)	50.7	42.1	-
Aß42 (>620 pg/ml)	1603	1290	-
Aß40	15747	12392	-
Ratio Aß42/40 (>0.06)	0.102	0.104	-
Blood brain barrier disturbance	0	0	-
Intrathecal IgG synthesis	0	0	-
autoantibodies	0	Anti-ARHGAP26 + 1:1	-
ASI		16.32	
**Serum**			
autoantibodies	0	Anti-ARHGAP26 ++ 1:32	Anti-ARHGAP26 ++ 1:100

Abbreviations: AI = antibody index, Aß40 = amyloid-ß 40, Aß42 = Amyloid-ß 42, ASI = antibody specific index, B-ADL = Bayer Activity of Daily Living Scale, CSF = cerebrospinal fluid, GDS = Geriatric Depression Scale, IgA = immunoglobulin A, IgG = immunoglobulin G, IgM = immunoglobulin M, MMSE = Mini Mental Status Examination, P Tau Protein 181 = phosphorylated tau protein 181, Q = Quotient, ratio Aß42/40 = ratio of amyloid-ß 42/ ratio of amyloid-ß 40, VZV = Varizella zoster virus, y = years. The values are depicted as mean ± standard deviation. For laboratory data normal ranges are shown in brackets. CSF analysis was done in the Neurochemistry Laboratory from the Department of Neurology, University Medical Center Göttingen. All cell destruction parameter were derived from the Laboratory for Clinical Neurochemistry and Dementia Diagnostics of the Department of Psychiatry and Psychotherapy, University Hospital Erlangen.
